# Incidence of primary hepatitis C infection and risk factors for transmission in an Australian prisoner cohort

**DOI:** 10.1186/1471-2458-10-633

**Published:** 2010-10-22

**Authors:** Suzy Teutsch, Fabio Luciani, Nicolas Scheuer, Luke McCredie, Parastu Hosseiny, William Rawlinson, John Kaldor, Gregory J Dore, Kate Dolan, Rosemary Ffrench, Andrew Lloyd, Paul Haber, Michael Levy

**Affiliations:** 1 School of Medical Sciences, The University of New South Wales, Sydney, New South Wales, Australia; 2 National Drug and Alcohol Research Centre, The University of New South Wales, Sydney, New South Wales, Australia; 3 Centre for Health Research in Criminal Justice, Justice Health, Sydney, New South Wales, Australia; 4 Virology Division, SEALS, Prince of Wales Hospital, Sydney, New South Wales, Australia; 5 The National Centre in HIV Epidemiology and Clinical Research, The University of New South Wales, Sydney, New South Wales, Australia; 6 The Burnet Institute, Melbourne, Victoria, Australia; 7 Drug Health Services, Royal Prince Alfred Hospital, Sydney, New South Wales, Australia; 8 College of Health Science, Australian National University, Canberra, Australian Capital Territory, Australia

## Abstract

**Background:**

Hepatitis C virus (HCV) infection is common in prisoner populations, particularly those with a history of injecting drug use (IDU). Previous studies of HCV incidence have been based on small case numbers and have not distinguished risk events in prison from those in the community.

**Methods:**

HCV incidence was examined in a longitudinal cohort of 488 Australian prisoners with a history of IDU and documented to be seronegative within 12 months prior to enrolment. Inmates were tested for anti-HCV antibodies and viremia, and interviewed about demographic and behavioral risk factors for transmission.

**Results:**

The cohort was predominantly male (65%) with high rates of prior imprisonment (72%) and tattooing (73%), as well as longstanding IDU (mean 8.5 years). Ninety-four incident HCV cases were identified (incidence 31.6 per 100 person years). Independent associations were observed between incident infection and prior imprisonment (*p *= 0.02) and tattooing (*p *= 0.03), and surprisingly also with methadone maintenance treatment (MMT) (*p *< 0.001).

**Conclusions:**

High rates of new HCV infection were found in this prisoner cohort reflecting their substantive risk behavior profile, despite having remained uninfected for many years. The association with MMT is challenging and highlights the need for better understanding of prison-specific HCV transmission risks, as well as the uptake and effectiveness of prevention programs.

## Background

Hepatitis C (HCV) is primarily transmitted through parenteral exposure to contaminated blood. Injecting drug use (IDU) with sharing of injecting equipment accounts for the majority of new infections in developed countries [1]. HCV has infected 2.2% of the world's population [2], with prevalence rates of up to 91% documented in IDU populations with injecting histories of more than five years, and incidence rates as high as 45.8 per 100 person-years recorded in new IDU [3,4].

Very close relationships exist between illicit drug use, HCV infection, and imprisonment [5]. Injecting drug users (IDUs) have high rates of imprisonment predominantly due to the criminalization of their drug use, and the tendency to fund some drug use through crime. For instance, almost half of all prison inmates in Australia report a lifetime history of IDU and approximately 70% have been incarcerated for drug-related crimes [6]. Imprisonment is an independent risk factor for HCV infection in community-based IDUs [7,8]. Estimates of the prevalence of IDU in prison have ranged from 11% in Canada [9] to 53% in Scotland [10].

Among IDU prisoners, HCV incident case rates range widely from less than 6% up to 38% per annum [11,12]. It should be noted that these estimates were all based on small numbers of incident cases (range: 1-14 cases; mean 7), and were largely unable to separately define the case rates associated with transmission events in prison from those occurring shortly prior to imprisonment or during periods of release to freedom. Incident HCV infection in prison inmates has been correlated with several demographic and behavioral risk factors, including age, female gender, tattooing, as well as specific injecting behaviors including sharing of needles and syringes and heroin as a drug of choice [13-18]. A recent meta-analysis of six published studies of HCV incidence in prison inmates [11,13,15,18-20] reported that the risk of HCV infection was eight times greater in IDU prisoners than in non-IDU prisoners [21].

The aims of this study were to determine HCV incidence amongst an IDU prisoner cohort delineating likely in-prison from community transmission events, and to carry out a detailed examination of demographic and behavioral risk factors associated with HCV transmission.

## Methods

### Subjects

From September 2005 to May 2009 adult prison inmates were recruited at 19 of 30 correctional centres housing adult male or females in New South Wales, Australia as part of the Hepatitis C Incidence and Transmission in Prisons Study (HITS-II) prospective cohort. The data reported here were derived from the enrolment interview and laboratory testing for anti-HCV antibodies and HCV RNA.

Male and female inmates were recruited into the HITS cohort by several methods, including promotional flyers at prison clinics, word of mouth amongst inmates, and by research nurses directly approaching inmates who had a recent negative anti-HCV antibody test result in their prison medical record (of whom less than 10% declined enrolment). Subjects were enrolled if they met all of the following inclusion criteria: 18 years of age or older; self-reported lifetime history of IDU; a documented negative anti-HCV antibody test result within the previous 12 months (performed either in prison or in the community, and sighted in the prison medical file); and adequate English to provide both valid written informed consent and to participate in the study interview. Subjects were excluded if they were forensic inmates (i.e. those unable to stand trial due to mental illness), or had HIV infection, chronic hepatitis B infection, or were pregnant at the time of enrolment (as these parameters impacted on immunological studies which form part of the study protocol reported elsewhere - Cameron B et al., manuscript in preparation). All subjects provided written, informed consent and received a $A10 payment upon completion of the interview and initial blood sampling. The consent form included specific details about the risk factors for acquiring hepatitis C and a listing of hepatitis services for contact should further information be required, as well as a provision for informing inmates of their HCV serology and virology test results and post-test counselling, with referral to a hepatitis clinical service for further assessment and care if the results suggested HCV infection.

### Interview

The interviews were conducted by a research nurse employed through Justice Health (i.e. the prison health service, which is independent of the custodial authority). The initial interview, adapted from that used previously in the HITS-I study [22] recorded demographic characteristics, and behavioral risk factors for HCV transmission (such as injecting behaviors, physical assaults or injuries, tattooing and body piercing) over: the lifetime; in the three months prior to current incarceration; and in the period during the current incarceration (see Additional file 1: Appendix).

### HCV serological and virological testing

A 10 ml venous blood sample was collected from each inmate. HCV antibody testing was performed using the qualitative Abbott ARCHITECT Anti-HCV chemiluminescent microparticle immunoassay (Abbott Diagnostics, Abbott Park, IL, USA), and confirmed either using the INNOTEST HCV Ab IV ELISA assay (Innogenetics, Gent, Belgium) or Monolisa™ HCV Ag-Ab ULTRA assay (Bio-Rad Laboratories, Marnes-la-Coquette, France). Qualitative HCV RNA detection was performed either using the VERSANT HCV RNA Qualitative Transcription Mediated Amplification (TMA) assay (Bayer Diagnostics, Emeryville, CA, USA; lower limit of detection: 3200 copies/ml) or COBAS AmpliPrep/COBAS TaqMan HCV assay (Roche, Branchburg, NJ, USA; lower limit of detection 223 copies/ml).

### Data management

All interviews and HCV test results were entered into a database created within SPSS for Windows (v. 17.0, Chicago, IL, USA). A random sample of entered data (10% of records) was checked by a study member not involved in data collection or data entry, with a larger data sample checked if the error rate was found to be greater than 5%. Data entry errors were corrected prior to statistical analyses.

### Statistical analysis

Unadjusted HCV incidence rates were determined using the person-years method with exact 95% Poisson confidence intervals [23]. Different intervals of observation were estimated according to HCV seroconversion status in order to reliably attribute risk behaviors for HCV infection either to within-prison or outside-prison: firstly for inmates who were HCV seropositive on the enrolment test (regardless of whether they were HCV RNA positive or negative), 42 days was added to the pre-enrolment anti-HCV antibody negative test date to account for the period prior to that date during which risk behaviors associated with HCV transmission may have occurred, but was not yet detected as seroconversion on the first test [24]. The estimated date of infection for these subjects was then taken as the midpoint between the adjusted first and second HCV test dates. Secondly, for inmates who were HCV seronegative and RNA negative on the enrolment test, the same adjustment for the first test date was made. Thirdly, for inmates who were HCV RNA positive on the enrolment test, but seronegative or antibody indeterminate on that date, 14 days was subtracted from the enrolment HCV test date to provide the estimated date of infection.

A corroborative incidence estimate was also calculated based on the number of subjects who were viremic, but seronegative. This estimate is based on an assumption that in primary infection, viremia precedes seroconversion by a median of 42 days, allowing incidence to be calculated from cross-sectional data [25], according to the following formula: I = (n­_vsn_/N_sn_) × (365/42) × (100); where I = incidence per 100 py, n-_vsn _= number of HCV RNA positive/seronegative subjects, N_sn _= total number of seronegative subjects.

Incidence rates within the following subgroups were determined: age at first test (18-25 years vs. >25 years), gender, whether Aboriginal and/or Torres Strait Islander (ATSI), whether continuously in prison for the duration of observation (defined as having been incarcerated at least 42 days prior to the initial HCV test until the time of the second test), injecting drugs since most recent entry into prison, having a tattoo, duration of injection at pre-enrolment test (<5 years, 5-10 years, 10+ years), predominant drug injected (heroin, methamphetamine, cocaine, methadone/buprenorphine), sharing injecting equipment, and whether currently receiving methadone maintenance treatment (MMT). HCV incidence rates within subjects who were of ATSI ethnicity were specifically examined, as this population is significantly over-represented within the Australian custodial system, and has a higher prevalence of blood borne viral infections [26,27]. Subjects were designated as ATSI on the basis of self-report during the enrolment interviews. In addition, incidence rates were determined for inmates who were not continuously in prison during the period of observation and who reported injecting drugs in the three months prior to imprisonment, as this may have contributed to incident infection within this sub-group.

Univariate associations between demographic and behavioral risk factors for HCV infection were performed using the χ^2 ^statistic for categorical data or unpaired two-tailed *t *tests for continuous data (SPSS for Windows v. 17.0). Risk factors with trends towards associations (*p *< 0.2) in the univariate analyses, in addition to those previously reported in the published literature were further investigated in multivariate logistic regression analysis using R [28] and SAS (v. 9.1; Cary, NC, USA) statistical software packages.

### Ethics

Ethical approval was obtained from Human Research Ethics Committees of Justice Health (reference number GEN 31/05), New South Wales Department of Corrective Services (reference number 05/0884), and the University of New South Wales (reference number 05094), all located in Sydney, Australia.

## Results

### Demographics

The demographic characteristics of the 488 inmates are shown in Table 1. The subjects were representative of the general IDU inmate population, including a predominance of young males (mean age 28 years) with relatively low educational attainment (76% with less than 10 years of education), and a high rate of previous imprisonment (72%) and longstanding IDU (mean duration 8.5 years) with a high frequency of sharing behavior (63%). Approximately one quarter of subjects reported ongoing IDU since imprisonment (n = 133; 27%).

**Table 1:** Demographic and behavioral characteristics of subjects enrolled in the HITS cohort (n = 488)

Variable	N = 488
Mean age; years (SD)	28 (6.9)
Male; No. (%)	318 (65)
Less than 10 years education; No. (%)	373 (76)
ATSI^†^; No. (%)	121 (25)
Non-English speaking background; No. (%)	9 (2)
Previous imprisonment; No. (%)	352 (72)
Ever had a tattoo; No. (%)	354 (73)
Mean duration of IDU; years (SD)	8.5 (6.2)
Ever shared injecting equipment; No. (%)	306 (63)
IDU whilst in prison; No. (%)	133 (27)

^† ^Aboriginal or Torres Strait Islander descent

### HCV incidence

Analysis of the enrolment blood samples identified 94 HCV incident cases (19% of the cohort) located in 17 of the 19 recruiting correctional centers, including 51 men (54%) and 43 women (46%). Eighty-five of these incident cases (90%) had seroconverted, of whom 57 (67%) were viremic; and another nine (10%) were viremic, but were either antibody indeterminate or negative.

The overall HCV incidence rate was 31.6 per 100 person years (100 py; 95% confidence interval 25.6-38.7). The incidence rate based on seropositive subjects only (N = 85) was 28.6 per 100 py (22.8-35.4). The corroborative incidence estimate based on the viremic, but seronegative, case rate was 16.0 per 100 py (7.4-30.0). Analysis of HCV incidence rates by selected demographic and behavioral subgroups (Table 2) showed that the HCV incidence was significantly higher in subjects who were female (*p *= 0.02), and somewhat higher in subjects with ATSI background, though this did not reach significance (*p *= 0.09). Incidence rates were significantly higher in those who reported: having been tattooed (*p *= 0.01); IDU in the three months prior to imprisonment (*p *< 0.001), and in those who were receiving MMT (*p *< 0.001), including those who reported injecting methadone (*p *= 0.05). Inmates who had been continuously in prison had a lower incidence (*p *= 0.04).

**Table 2:** HCV incidence rates in the HITS cohort stratified for demographic and behavioral risk variables (N = 488)

	No. incident cases	**Incidence rate per 100 p.y**.	**95% CI**^**†**^	**Rate ratio**^§^	**95% CI**^**††**^
**Total sample**	94	31.6	25.6 - 38.7		
**Age at first HCV test**					
18 - 25 years	35	26.4	18.4 - 36.6	1.0	
>25 years	59	35.9	27.3 - 46.3	1.4	0.9 - 2.1
**Gender**					
Male	51	26.2	19.5 - 34.4	1.0	
Female	43	42.0	30.4 - 56.5	**1.6**	1.1 - 2.4
**Ethnic background**					
Other	68	29.2	22.7 - 37.0	1.0	
ATSI	26	40.4	26.4 - 59.2	1.4	0.9 - 2.2
**Continuously in prison**					
No	70	37.0	28.8 - 46.7	1.0	
Yes	24	22.6	14.5 - 33.6	**0.6**	0.4 - 1.0
**Ever had a tattoo**					
No	17	19.3	11.2 - 30.8	1.0	
Yes	76	36.5	28.7 - 45.6	**1.9**	1.1 - 3.3
IDU in 3 months prior to imprisonment*					
No	36	20.9	14.6 - 28.9	1.0	
Yes	58	46.6	35.4 - 60.2	**2.2**	1.5 - 3.4
**IDU since imprisonment**					
No	63	29.3	22.5 - 37.5	1.0	
Yes	31	38.0	25.8 - 54.0	1.3	0.83 - 2.0
**Duration of IDU**					
<5 years	21	24.3	15.0 - 37.1	1.0	
5 - 10 years	43	36.6	26.5 - 49.3	1.5	0.9 - 2.6
>10 years	29	31.3	21.0 - 45.0	1.3	0.7 - 2.3
**Main drug injected**					
Heroin	69	35.1 ???.4	27.3 - 44.5	1.4	0.9 - 2.3
Methamphetamine	82	32.3	25.7 - 40.1	1.1	0.7 - 2.2
Cocaine	48	34.0	25.1 - 45.1	1.2	0.8 - 1.7
Methadone/buprenorphine	40	40.7	29.1 - 55.5	**1.5**	1.0 - 2.3
**Sharing injecting equipment**					
No	31	30.0	20.3 - 42.5	1.0	
Yes	63	33.8	26.0 - 43.3	1.1	0.7 - 1.8
**Receiving MMT**					
No	58	24.6	18.6 - 31.7	1.0	
Yes	36	60.1	42.1 - 83.2	**2.5**	1.6 - 3.7

^† ^Poisson-exact 95% confidence intervals

^§ ^Figures in bold are significant at p < 0.05

^**††**. ^Exact method (mid-p)

* Only among inmates who were not continuously in prison included (n = 246)

### Demographic and behavioral risk associations with HCV incident infection

Univariate analyses examined associations between HCV incidence and demographic or behavioral risk variables recorded in relation to the following time periods: 'ever'; 'in the three months prior to entry into prison', and 'since entry into prison' (Table 3). Significant positive associations were observed between incident HCV infection and: a shorter duration of current imprisonment (mean 28 versus 52 weeks; *p *< 0.001); a higher frequency of previous imprisonment (84% of incident cases versus 70% of non-cases; *p *< 0.01); a longer duration of IDU (mean 9.5 versus 8.1 years; *p *= 0.04); ever having had a tattoo (81% of incident cases versus 71% of non-cases, *p *= 0.03), female gender (46% versus 32%, *p *= 0.01), ever having ever injected drugs either in prison or in a juvenile detention center (52% versus 37%, *p *= 0.01), having ever had an injecting frequency of daily or more (87% versus 75%, *p *= 0.01); and receiving MMT at the time of the interview (38% versus 16%; *p *< 0.001). Injecting methadone was also significantly associated with incident infection (43% vs. 32%, p = 0.046). Having injected drugs (83% versus 65%, *p *< 0.01); injected daily or more often (71% versus 43%, p < 0.001); and having always bleached shared equipment (19% versus 9%, *p *= 0.04) in the three months prior to incarceration were also positively associated. Negative associations were also observed, including continuous imprisonment during the period of observation (26% versus 55%, p < 0.001), having had a recent break from IDU (i.e. six months or more without reported injecting) (35% versus 53%, *p *= 0.002), and having been injected by someone else (62% versus 72%, *p *= 0.04).

**Table 3:** Univariate analyses of demographic and risk behavior for incident HCV infection in the HITS cohort (n = 488)

Variable	Incident cases (n = 94)	Uninfected subjects (n = 394)	*p*	95% CI
Mean age; years (SD)	28 (±7)	27 (±7)	0.08	
Male gender; No. (%)	51 (54)	267 (68)	**0.01**	0.4 - 0.9
ATSI; No. (%)	26 (28)	92 (23)	0.38	0.8 - 2.1
Less than 10 years education; No. (%)	71 (76)	302 (77)	0.82	0.6 - 1.6
Duration of imprisonment; weeks (SD)	28 (±39)	52 (±95)	**<0.001**	
Previously imprisoned; No. (%)	79 (84)	276 (70)	**<0.01**	1.2 - 4.1
Continuous imprisonment; No. (%)	24 (26)	216 (55)	**<0.001**	0.2 - 0.5
**Ever**:				
Mean duration of IDU; years (SD) (mean, years)	9.5 (±6.8)	8.1 (±6.0)	**0.04**	
Tattoo; No. (%)	76 (81)	278 (71)	**0.03**	1.1 - 3.3
Body-piercing; No. (%)	68 (72)	307 (78)	0.25	0.4 - 1.2
Fight with blood; No. (%)	37 (39)	167 (42)	0.79	0.6 - 1.5
Stabbed; No. (%)	24 (26)	109 (28)	0.68	0.5 - 1.5
Shared a razor; No. (%)	12 (13)	66 (17)	0.39	0.4 - 1.5
Haircut with laceration; No. (%)	21 (22)	85 (22)	0.87	0.6 - 1.8
Blood contact during sport; No. (%)	12 (13)	80 (20)	0.11	0.3 - 1.1
Needle-stick injury; No. (%)	21 (22)	68 (17)	0.28	0.8 - 2.4
IDU in prison; No. (%)	49 (52)	147 (37)	**0.01**	1.1 - 2.8
Injected by someone else; No. (%)	58 (62)	284 (72)	**0.04**	0.4 - 1.0
Injected heroin; No. (%)	69 (73)	249 (63)	0.06	1.0 - 2.7
Injected methamphetamine; No. (%)	82 (87)	341 (87)	0.86	0.5 - 2.1
Injected methadone/buprenorphine; No. (%)	40 (43)	125 (32)	**0.05**	1.0 - 2.5
Daily IDU; No. (%)	82 (87)	296 (75)	**0.01**	1.2 - 4.3
Shared IDU equipment; No. (%)	63 (67)	243 (62)	0.43	0.8 - 2.0
Drug & Alcohol counseling; No. (%)	69 (73)	303 (77)	0.42	0.5 - 1.4
3 months prior to incarceration*				
IDU; No. (%)	58 (83)	115 (65)	**<0.01**	1.3 - 5.1
Daily IDU; No. (%)	50 (71)	75 (43)	**<0.001**	1.9 - 6.1
Shared IDU equipment; No. (%)	18 (26)	41 (23)	0.69	0.6 - 2.2
Always bleach shared equipment, No. (%)	13 (19)	16 (9)	**0.04**	1.0 - 5.0
**Since incarceration**:				
IDU; No. (%)	31 (33)	102 (26)	0.17	0.9 - 2.3
Daily IDU; No. (%)	3 (3)	15 (5)	0.56	0.2 - 2.4
Shared IDU equipment; No. (%)	27 (29)	93 (24)	0.30	0.8 - 2.2
Always bleach shared equipment, No. (%)	20 (21)	63 (16)	0.22	0.8 - 2.5
Recent break from IDU >6 months; No. (%)	33 (35)	208 (53)	**<0.01**	0.3 - 0.7
Currently on MMT; No. (%)	36 (38)	63 (16)	**<0.001**	2.0 - 5.3

* Only inmates who were not continuously in prison were included in this analysis (n = 246)

The results of the multivariate logistic regression analysis are shown in Table 4. Independent positive associations were observed between HCV incident infection and receiving MMT (*p *< 0.001) and previous imprisonment (*p *= 0.02). Positive associations were also observed with having tattoos (*p *= 0.03), and with a frequency of injecting of daily or more (*p *= 0.02) in the three months prior to imprisonment.

**Table 4:** Associations between demographic and behavioral characteristics and HCV incident case status using logistic regression analysis (n = 488)

Variable	Odds ratio	95% CI	*p*
Age >25 years	1.29	0.69 - 2.43	0.43
Male gender	0.75	0.41 - 1.39	0.37
ATSI	1.36	0.73 - 2.53	0.33
Previously imprisoned	2.45	1.18 - 5.11	**0.02**
Continuously in prison	0.68	0.27 - 1.62	0.41
Duration of imprisonment (weeks)	0.93	0.73 - 1.20	0.60
Ever had a tattoo	2.01	1.01 - 4.01	**0.05**
Duration of IDU (years)	1.27	0.84 - 1.92	0.26
Ever shared IDU equipment	1.34	0.69 - 2.62	0.39
Ever daily IDU	0.77	0.34 - 1.76	0.53
Ever injected heroin	0.76	0.39 - 1.48	0.42
Ever injected methadone/buprenorphine	1.20	0.63 - 2.29	0.57
Ever IDU in prison	0.87	0.37 - 2.04	0.74
Ever injected by someone else	0.62	0.34 - 1.14	0.12
IDU in 3 months prior to imprisonment*	0.93	0.31 - 2.78	0.89
Daily IDU in 3 months prior to imprisonment*	3.70	1.36 - 10.10	**0.01**
Always bleached IDU equipment in 3 months prior to imprisonment*	0.38	0.1 - 1.39	0.14
IDU since imprisonment	1.87	0.37 - 9.46	0.45
Shared IDU equipment since imprisonment	0.91	0.18 - 4.48	0.91
Recent break from IDU (>6 months)	0.90	0.47 - 1.70	0.74
Receiving MMT	3.13	1.66 - 5.91	**<0.001**

* Only inmates who were not continuously in prison were included in this analysis (n = 246)

In order to further explore the unexpected association between incident infection and receiving MMT, the IDU behaviors reported by inmates receiving MMT who became incident cases and non-cases were compared with those not receiving MMT (see Additional file 2). The total group of inmates receiving MMT was more likely to report having ever injected daily than those not receiving MMT (*p *= 0.001); IDU since entry into prison (*p *= 0.04); and sharing IDU equipment since entry into prison (*p *= 0.08), suggesting more significant opioid dependency (and hence the indication for MMT). Those receiving MMT were also more likely to report a 'decreasing' pattern of IDU since entry into prison, than those not receiving MMT (*p *= 0.01), suggesting the treatment was likely to be favoring a reduction in injecting behavior of those on treatment. However, inmates receiving MMT were more likely to report injecting methadone than those not receiving MMT (52% vs. 29%, *p *< 0.001), but this report was in relation to their lifetime pattern, and was not continued while imprisoned (*p *= 0.81).

## Discussion

This study reports the largest number of incident HCV cases identified in a longitudinal cohort of prison inmates (n = 94). The overall HCV incidence rate (31.6 per 100 py) was higher than most previous reports [11-15,18], potentially reflecting the specific inclusion of inmates who reported a lifetime history of IDU. This substantive case rate is noteworthy as the enrolled population can reasonably be categorized as longstanding and high risk IDUs with a mean duration of injecting (8.5 years), and with sharing of IDU equipment (63%), high rates of tattooing (73%), and prior imprisonment (72%) - each of which has been independently associated with the risk of HCV infection. Thus, this group appears to have been at high risk of HCV infection over a prolonged period, but have remarkably remained seronegative, and yet have a high rate of incident infection associated with incarceration. This high incidence may relate to changes in specific IDU behaviors in the unstable period both prior to, and immediately following, incarceration. Indeed, in both the univariate and logistic regression analyses, stronger associations were found with risk behaviours, such as IDU and sharing reported in the three months prior to imprisonment, than those 'since imprisonment'. Further, it is plausible that there is a causative relationship relating to drug availability, imprisonment and incident infection in that increasing drug dependency and/or variations in drug supply may prompt the illegal acts which lead to arrest and imprisonment, and incident HCV infection in this period.

The cohort included IDU inmates who had been continuously imprisoned and those who had been both outside and inside prison during the period of surveillance. A preliminary analysis of a subset of the inmates reported here, who were continuously in prison (n = 120) has been described elsewhere [29], which revealed an incidence of 34.2 per 100 py. This estimate is somewhat higher than the incidence of those continuously imprisoned in the current analysis (22.6 per 100 py).

In previous studies of IDU in the community, predictors of HCV seroconversion have included: reporting injecting in the previous six months, and more than daily IDU [3]; a duration of injecting of less than one year and injecting cocaine [30,31], sharing needles [3,30,32] and sharing other injecting paraphernalia [3,32,33], being of female gender, and having a culturally and linguistically diverse background (CALD) [31]. Among new IDUs with a CALD background in Australia, HCV seroconversion was strongly associated with sharing syringes, and other injecting equipment [31]. By contrast, the only previous study of IDU prisoners with sufficient numbers of incident cases to allow risk assessment, revealed associations with older age, tattooing in prison, and injecting heroin [16]. Perhaps surprisingly therefore, this study found modest associations between the incidence of HCV infection and specific IDU behaviors in prison inmates with only IDU in the three months prior to imprisonment being associated with incident infection. The possible explanations for this outcome may firstly include under-reporting of actual IDU and sharing behaviors due to the difficulties in accurate recollection of specific IDU episodes over several months prior to the interview - this may be considered unlikely as more than half of the group reported daily IDU. Secondly, there may be limitations in the reporting of information relating to risk behaviors occurring in prison, as both IDU and tattooing are illegal - nevertheless the interviews were conducted in private and by research staff employed in the Justice Health Service, which is independent of the custodial authority.

Although consistent with previous reports [7,8,18], the associations between incident infection and lifetime histories of prior imprisonment and tattooing reported here, are unlikely to be direct as the subjects were found to be seronegative at screening. By contrast, it is likely that these variables act as surrogates for more recent risk behaviors potentially associated with HCV transmission. Thus, it is possible that there are additional risk behaviors relating to IDU (such as back-loading [4]), or other blood-to-blood events not adequately explored in the interview schedule. For instance, the enrolment interview did not include detailed investigation of recent tattooing episodes, fights, and other parenteral blood contacts. These events are being sought in the follow-up interviews in the HITS cohort.

The strong association observed in the data reported here between HCV incidence and MMT has not previously been reported. This association may be attributable to injecting the dispensed drug rather than ingesting the dose (despite being supervised), as the rates of reported injecting of methadone were significantly higher in incident cases compared to non-cases. It was also noteworthy that approximately half of the inmates receiving MMT reported a lifetime pattern of injecting methadone, whereas only approximately a third of those not receiving MMT reported this behavioral risk. However, this report was not sustained in prison, raising the possibility of under-reporting of methadone injecting by those receiving MMT whilst in prison.

Another limitation in relation to this finding is that methadone treatment status at the time of the initial negative HCV test result may not have been the same as that recorded at enrolment. It is possible that the incident cases (n = 36) and non-cases (n = 63) on MMT differed in the continuity of that treatment and hence the likelihood of associated risk behaviors, although the mean methadone dose and the reported number of missed doses did not differ between these subgroups (data not shown). It is also possible that MMT uptake in prison is greater among individuals who are at higher risk of HCV infection. Consistent with previous reports [16,17], there was evidence that MMT was generally associated with a reduced frequency of IDU and sharing. The positive association between MMT and HCV incidence therefore warrants further investigation. For instance, continuous recording of dosages and periods of cessation of MMT may be informative. The generalizability of the MMT association finding should also be taken with caution, given the likely variations in HCV incidence and clear differences in MMT policies in prison settings internationally [34].

One of the mainstays of the prevention strategies for blood borne virus transmission in this prison setting is the provision of bleach (1% hypochlorite solution) and a recommended protocol (a repeated water rinse following by repeated bleach rinse, followed by repeated water rinse) for cleansing of injecting apparatus. Previous reports have raised concerns that although this disinfection method is likely to reduce HIV transmission, it may be less effective for HCV [14,35-38]. Consistent with this concern, there was no apparent reduction in HCV incidence among individuals who reported IDU and sharing, but who indicated that they always bleach-cleansed the injecting apparatus.

## Conclusions

The findings reported here raise concerns that imprisonment of IDUs is associated with an unacceptably high risk of acquisition of HCV infection, in particular in the period surrounding imprisonment. Further investigation of the specific circumstances in which transmissions are occurring is warranted to guide effective prevention strategies, such as introduction of professional tattooists and needle-syringe exchange programs in prison.

## Abbreviations

HCV: hepatitis C virus; IDU: injecting drug use; MMT: methadone maintenance treatment; ATSI: Aboriginal or Torres Strait Islander.

## Competing interests

The authors declare that they have no competing interests.

## Authors' contributions

ST and ML led the writing of the manuscript and the initial data analysis. FL and NS ran the majority of the data analyses with input from AL, GD, KD, and PH. AL, ML, PH, KD, WR, JK, RF contributed to the overall conception, design and interpretation of the study, and to the writing. FL, NS, GD, LM, and PH contributed to the writing. All authors read and approved the final manuscript.

## Pre-publication history

The pre-publication history for this paper can be accessed here:

http://www.biomedcentral.com/1471-2458/10/633/prepub

## Supplementary Material

Additional file 1 **Appendix - Hepatitis C Incidence and Transmission in Prisons Study (HITS-II) Enrolment Interview**. Questionnaire document Click here for file

Additional file 2 **IDU behaviors amongst inmates receiving MMT**. Table of frequencies and univariate comparisons Click here for file
