# Supplementary material for: Incidence of primary hepatitis C infection and risk factors for transmission in an Australian prisoner cohort
Source: BMC Public Health. 2010 Oct 22;10:633. doi: 10.1186/1471-2458-10-633 (PMC2975656; doi:10.1186/1471-2458-10-633)
Supplement: Additional file 1 — Appendix - Hepatitis C Incidence and Transmission in Prisons Study (HITS-II) Enrolment Interview. Questionnaire document [file 1471-2458-10-633-S1.DOC]

**Appendix**

Hepatitis C Incidence and Transmission in Prisons Study (HITS-II) Enrolment Interview

**Hepatitis C Incidence and Transmission S**tudy (HITS)-II

| **2x2 Code** (Surname first**)** |  | | |  | | |  | | | |  | | |
| --- | --- | --- | --- | --- | --- | --- | --- | --- | --- | --- | --- | --- | --- |
| **Date of Birth** | **D** | | **D** | | | **M** | | **M** | | **Y** | | | **Y** |
| **MIN** |  |  | | |  | |  | |  | | |  | |
| **Current Prison** [Interviewer – see code on page 2] | | | | | | | | | | | | | |
| **Interviewers Initials** | | | |  | | |  | | | |  | | |

**Serology: (**ensure that both the Date and Positive/Negative/Unknown fields are completed)

| *HIV, HCV, HBV status* | **Date** | **Positive** |  | **Negative** |  | **Unknown** |  |
| --- | --- | --- | --- | --- | --- | --- | --- |
| HIV Ab |  |  | 1 |  | 2 |  | 9 |
| HbcAb (core antibody) |  |  | 1 |  | 2 |  | 9 |
| HbsAb (surface antibody) |  |  | 1 |  | 2 |  | 9 |
| HbsAg (surface antigen) |  |  | 1 |  | 2 |  | 9 |
| HCV Ab |  |  | 1 |  | 2 |  | 9 |
| HCV PCR |  |  | 1 |  | 2 |  | 9 |

| **Eligibility criteria (**tick relevant boxes) | **Yes** |  | **No** |  | **Unknown** |  |
| --- | --- | --- | --- | --- | --- | --- |
| Age 18 years or greater |  | 1 |  | 2 |  | 9 |
| IDU |  | 1 |  | 2 |  | 9 |
| Been in prison less than 12 months |  | 1 |  | 2 |  | 9 |

| **Enrolment Criteria (**tick relevant boxes) | **Yes** |  | **No** |  | **Date** |
| --- | --- | --- | --- | --- | --- |
| Signed Consent form |  | 1 |  | 2 |  |
| Subject recruited? |  | 1 |  | 2 |  |
| Intake Interview completed? |  | 1 |  | 2 |  |
| Follow up Contact Form completed? |  | 1 |  | 2 |  |
| Blood taken? |  | 1 |  | 2 |  |

**Current Prison** (please tick relevant box)

| Bathurst |  | 1 | Kirkconnell |  | 17 |
| --- | --- | --- | --- | --- | --- |
| Berrima |  | 2 | Lithgow |  | 18 |
| Bolwara Transitional Centre |  | 3 | Long Bay Complex |  | 19 |
| Brewarrina |  | 4 | Maitland |  | 20 |
| Broken Hill |  | 5 | Mannus |  | 21 |
| Cessnock |  | 6 | Mid North Coast |  | 22 |
| Cooma |  | 7 | MRRC |  | 23 |
| Dilwynnia |  | 8 | Mulawa |  | 24 |
| Emu Plains |  | 9 | Norma Parker |  | 25 |
| Glen Innes |  | 10 | Oberon |  | 26 |
| Goulburn |  | 11 | Parklea |  | 27 |
| Grafton |  | 12 | Parramatta |  | 28 |
| John Morony I |  | 13 | Silverwater |  | 29 |
| John Morony II |  | 14 | St.Heliers |  | 30 |
| Junee |  | 15 | Tamworth |  | 31 |
| Ivanhoe |  | 16 |  |  |  |
|  |  |  |  |  |  |
|  |  |  |  |  |  |
|  |  |  |  |  |  |

| I. THIS SECTION SHOULD BE COLLECTED FROM OIMS/MEDICAL RECORD |
| --- |

|  | On reception was the client assessed to be intoxicated? | Yes (Go to Q 2) |  | 1 |
| --- | --- | --- | --- | --- |
|  |  | No (Go to Q 3) |  | 2 |
|  |  | Unknown |  | 9 |
|  |  |  |  |  |
|  | What substance caused the intoxication? | _____________________ |  |  |
|  |  | Unknown |  | 9 |
|  |  |  |  |  |
|  | When was the inmate arrested this time? | ____/____/____ |  |  |
|  |  | Don’t Know |  | 9 |
|  |  |  |  |  |
|  | When did inmate come into prison this time? | ____/_____/____ |  |  |
|  |  | Don’t Know |  | 9 |
|  |  |  |  |  |
|  | Has the inmate been sentenced? | Yes (go to 6) |  | 1 |
|  |  | No (go to 7) |  | 2 |
|  |  | Don’t know (go to 7) |  | 9 |
|  |  |  |  |  |
|  | What is the total length of this sentence? | __________MONTHS |  |  |
|  |  | Don’t know |  | 9 |
|  |  |  |  |  |
|  | What is inmate’s current security  classification? | ____________________ |  |  |
|  |  |  |  |  |
|  | [*Interviewer: see security classification code*] |  |  |  |
|  |  |  |  |  |
|  | How many times has the inmate been in an adult prison including this episode? | _______________ |  |  |
|  |  | Don’t Know |  | 9 |
|  |  |  |  |  |
|  | What is the most serious offence that the inmate is in prison for this time? | ______________________ |  |  |
|  |  |  |  |  |
|  | [*Interviewer: see offence code*] |  |  |  |

**Instructions for interviewer:**

- Complete all questions, unless instructed to go on to a specific question.
- Read the question exactly as it appears.
- For all questions, await a response from the subject, then code according to the responses listed.
- If the subject does not understand the question, repeat the question and list all of the responses before requesting a response from the subject.
- Most questions require you to tick the box next to the appropriate response number. Those with line require an answer to be written by you.
- Comments in *italics* explain aspects of the question.
- Don’t let the inmate know that by choosing option “NO” they can skip a section, since it may increase the chance of lying to finish off quickly.

| ***II. WE WILL START WITH SOME BACKGROUND INFORMATION*** [INTERVIEW] | | | | | | | | | |
| --- | --- | --- | --- | --- | --- | --- | --- | --- | --- |
|  |  |  | | | | | |  |  |
|  | What is your gender? | Male | | | | | |  | 1 |
|  |  | Female | | | | | |  | 2 |
|  |  | Transgender | | | | | |  | 3 |
|  |  |  | | | | | |  |  |
|  | Are you an Aboriginal? | Yes | | | | | |  | 1 |
|  |  | No | | | | | |  | 2 |
|  |  | Don’t Know | | | | | |  | 9 |
|  |  |  | | | | | |  |  |
|  | Are you a Torres Strait Islander? | Yes | | | | | |  | 1 |
|  |  | No | | | | | |  | 2 |
|  |  | Don’t Know | | | | | |  | 9 |
|  |  |  | | | | | |  |  |
|  | What country were you born in? | ___________________ | | | | | |  |  |
|  |  |  | | | | | |  |  |
|  | a. What country was your father born in?  *[Interviewer: If unknown code (9) in the provided space]* _________________ | | | | | | |  |  |
|  | b. What country was your mother born?  *[Interviewer: If unknown code (9) in the provided space]* _________________ | | | | | | |  |  |
|  |  |  | | | | | |  |  |
|  | What language do you speak at home? | English | | | | | |  | 1 |
|  |  | Other_________________ | | | | | |  | 2 |
|  |  |  | | | | | |  |  |
|  | Have you ever been in a juvenile detention centre? | Yes | | | | | |  | 1 |
|  |  | No | | | | | |  | 2 |
|  |  |  | | | | | |  |  |
|  | Were you using, or withdrawing from, an illegal drug when you committed the offence for which you are in prison? | Yes | | | | | |  | 1 |
|  |  | No | | | | | |  | 2 |
|  |  | Don’t Recall | | | | | |  | 9 |
|  |  |  | | | | | |  |  |
|  | Would you describe yourself as: | Straight | | | | | |  | 1 |
|  |  | Gay/Lesbian | | | | | |  | 2 |
|  |  | Bisexual | | | | | |  | 3 |
|  |  |  | | | | | |  |  |
|  | How many years of schooling have you completed? *[Interviewer: for answers like* | No formal education | | | | | |  | 0 |
|  |  | 1-6 years at school | | | | | |  | 1 |
|  | *“intermediate school”, or “junior high”, ask the* | 7-10 years at school | | | | | |  | 2 |
|  | *number of the years and tick the appropriate box.* | 11-12 years at school | | | | | |  | 3 |
|  |  | TAFE | | | | | |  | 4 |
|  | Tertiary Education | | | | | | |  | 5 |
|  |  |  | | | | | |  |  |
| III. THIS SECTION IS ABOUT RISK FACTORS FOR SPREAD OF HEP C [*Interviewer: all unsure responses, code as “Don’t recall”]* | | | | | | | | | |
|  |  |  | | | | | |  |  |
|  | Have you ever had a tattoo? | Yes (Go to 21) | | | | | |  | 1 |
|  |  | No (Go to 23) | | | | | |  | 2 |
|  |  | Don’t recall (Go to 23) | | | | | |  | 9 |
|  |  |  | | | | | |  |  |
|  | How many different times have you been tattooed? | __________________ | | | | | |  |  |
|  | [*Interviewer: consider each session of tattooing as a separate tattoo*] | | | | | | |  |  |
|  | *[Interviewer: Include tattoos that have been removed]* | | | | | | |  |  |
|  | Were the tattoos done inside or outside of prison or a juvenile detention centre? | Inside | | | | | |  | 1 |
|  |  | Outside | | | | | |  | 2 |
|  |  | Both Inside and outside | | | | | |  | 3 |
|  |  | Don’t know | | | | | |  | 9 |
|  |  |  | | | | | |  |  |
|  | Have you ever had any part of your body pierced? [*Interviewer: must mention that body* | Yes (go to 24) | | | | | |  | 1 |
|  |  | No (go to 26) | | | | | |  | 2 |
|  | *piercing include ear piercing as well*] | Don’t recall (go to 26) | | | | | |  | 9 |
|  |  |  | | | | | |  |  |
|  | How many times have you had your body pierced?  [*interviewer: include piercings that do not currently have a ring in them*] ______________ | | | | | | | | |
|  | [*Interviewer: if only ears are pierced, write the total number of the piercing in both ears,* | | | | | | | | |
|  | *i.e.: 1 piercing each ear = 2 in total]* | | | | | | | | |
|  |  |  | | | | | |  |  |
|  | Were the piercings done inside or outside of a prison or a juvenile detention centre? | Inside | | | | | |  | 1 |
|  |  | Outside | | | | | |  | 2 |
|  |  | Both inside and outside | | | | | |  | 3 |
|  |  | Don’t recall | | | | | |  | 9 |
|  |  |  | | | | | |  |  |
|  | Did you have a blood transfusion prior to 1990? | Yes | | | | | |  | 1 |
|  |  | No | | | | | |  | 2 |
|  |  | Don’t recall | | | | | |  | 9 |
|  |  |  | | | | | |  |  |
|  | Have you ever been in a fight where blood from another person may have come in contact with your mouth, eyes or an open wound? | Yes | | | | | |  | 1 |
|  |  | No | | | | | |  | 2 |
|  |  | Don’t recall | | | | | |  | 9 |
|  |  |  |  |  |  |  |  |  |  |
|  |  |  | | | | | |  |  |
|  | Have you ever been stabbed? | Yes | | | | | |  | 1 |
|  |  | No | | | | | |  | 2 |
|  |  | Don’t recall | | | | | |  | 9 |
|  |  |  | | | | | |  |  |
|  | Have you ever had a haircut where your skin or scalp was cut? | Yes | | | | | |  | 1 |
|  |  | No | | | | | |  | 2 |
|  |  | Unsure/Don’t recall | | | | | |  | 9 |
|  |  |  | | | | | |  |  |
|  | Have you ever shared the same razor as someone else? | Yes | | | | | |  | 1 |
|  |  | No | | | | | |  | 2 |
|  |  | Don’t recall | | | | | |  | 9 |
|  |  |  | | | | | |  |  |
|  | Have you ever had someone else’s blood on you during sport? (e.g. during a football game) | Yes | | | | | |  | 1 |
|  |  | No | | | | | |  | 2 |
|  |  | Don’t recall | | | | | |  | 9 |
|  |  |  | | | | | |  |  |
|  | Have you ever been accidentally pricked by a needle? (e.g. needle stick injury) | Yes | | | | | |  | 1 |
|  |  | No | | | | | |  | 2 |
|  | [*Interviewer: do not include IDU*] | Don’t recall | | | | | |  | 9 |
|  |  |  | | | | | |  |  |
| **IV. THIS IS A SECTION ABOUT DRUG USE** [*Interviewer: must tell the inmates that, the same questions about drug use are referring to different time of your life*] | | | | | | | | | |
|  |  |  | | | | | |  |  |
|  | Have you ever used illegal drugs? | Yes | | | | | |  | 1 |
|  |  | No | | | | | |  | 2 |
|  |  | Don’t recall | | | | | |  | 3 |
|  |  |  | | | | | |  |  |
|  | Have you ever-injected drugs? | Yes (go to Q35) | | | | | |  | 1 |
|  |  | No (go to Q 85) | | | | | |  | 2 |
|  |  | Don’t recall (go to Q 85) | | | | | |  | 3 |
|  |  |  | | | | | |  |  |
|  | Which of the following drugs have you injected? *[interviewer: read the options and emphasize on “injecting”]* |  | | | | | |  |  |
|  |  | Heroin | | | | | |  | 1 |
|  |  | Buprenorphine /Methadone | | | | | |  | 2 |
|  | Crystal meth/shabu/ice/goey/Amphetamine/Speed/methamphetamine | | | | | | |  | 3 |
|  | GHB/GBH/ liquid e/fantasy | | | | | | |  | 4 |
|  | *[interviewer: Exclude medications prescribed* | Cocaine/ Coke | | | | | |  | 5 |
|  | *and administered by health workers,* | Benzodiazepines/Benzos | | | | | |  | 6 |
|  | *e.g. Morphine in hospital* | Anabolic/Steroids | | | | | |  | 7 |
|  | Other opiates/morphine/pethidine/omnopon | | | | | | |  | 8 |
|  | Hallucinogens/LSD/ Acid/Magic/Mushies/Daitura | | | | | | |  | 9 |
|  |  | Ecstacy/ E/MDA/MDMA | | | | | |  | 10 |
|  |  | Ketamine | | | | | |  | 11 |
|  |  |  | | | | | |  |  |
|  | How old were you when you first injected drugs? | ____________ Years | | | | | |  |  |
|  |  | Don’t recall | | | | | |  | 9 |
|  |  |  | | | | | |  |  |
|  | Think about the period when you were injecting most frequently, which one of the following best describes how frequent that was? [*Interview: “injecting” refers to IDU*] | Less than monthly | | | | | |  | 1 |
|  |  | Monthly or more often | | | | | |  | 2 |
|  |  | Weekly or more often | | | | | |  | 3 |
|  |  | Daily | | | | | |  | 4 |
|  | *[Interviewer: check that “daily” and “ more than* | More than once a day | | | | | |  | 5 |
|  | *once a day” are distinguished]* | Don’t recall | | | | | |  | 9 |
|  |  |  | | | | | |  |  |
|  | Have you ever injected drugs in prison or a juvenile detention centre? | Yes | | | | | |  | 1 |
|  |  | No | | | | | |  | 2 |
|  |  | Don’t recall | | | | | |  | 9 |
|  |  |  | | | | | |  |  |
|  | Has someone else ever injected you with drugs? (Given you a hit?) | Yes | | | | | |  | 1 |
|  |  | No | | | | | |  | 2 |
|  | [*Interviewer: do not include medical injections*] | Don’t recall | | | | | |  | 9 |
|  |  |  | | | | | |  |  |
|  | Have you ever shared any part of the  injecting equipment? That includes the  needle, syringe, spoon, swabs, filters, mix  or tourniquet. | Yes | | | | | |  | 1 |
|  |  | No | | | | | |  | 2 |
|  |  | Don’t recall | | | | | |  | 9 |
|  |  |  |  |  |  |  |  |  |  |
|  | [*Interviewer : mention that this includes sharing with their partner, and even once is a “yes”,*] | | | | | | | | |
|  |  |  | | | | | |  |  |
|  |  |  | | | | | |  |  |
| V. THIS SECTION IS ABOUT TAKING BREAK FROM INJECTING [*Interviewer: “Break” is absolutely NO injecting*] | | | | | | | | | |
|  |  |  | | | | | |  |  |
|  | In the last 12 months have you had a break from injecting? | Yes (Go to Q 42) | | | | | |  | 1 |
|  |  | No (Go to Q 43) | | | | | |  | 2 |
|  |  | Don’t recall (Go to Q 43) | | | | | |  | 9 |
|  |  |  | | | | | |  |  |
|  | How long was the longest break? | Less than one month | | | | | |  | 1 |
|  |  | 1- 3 months | | | | | |  | 2 |
|  |  | 3- 6 months | | | | | |  | 3 |
|  |  | 6-9 months | | | | | |  | 4 |
|  |  | 9-12 months | | | | | |  | 5 |
|  |  | More than 12 months | | | | | |  | 6 |
|  |  |  | | | | | |  |  |
| ***VI. THE NEXT SECTION REFERS TO DRUG USE IN THE 3 MONTHS BEFORE COMING INTO PRISON***  [*Interviewer: Check OIMS or by asking the inmate to clarify if this is the first imprisonment*] [*Interviewer: if the inmate been outside the prison less than 3 months Q43-Q64 should refer just to that period*] | | | | | | | | | |
|  |  | | | | | | | | |
| 43. | How long were you out before coming in to prison this time? | 3 months or more | | | | | |  | 1 |
|  |  | _____________________ | | | | | |  |  |
|  | *[Interviewer: If less than 3 months, write the duration and adjust the questions accordingly]* |  | | | | | |  |  |
|  |  |  | | | | | |  |  |
|  | In the 3 months *[or as appropriate]* before coming into prison did you inject drugs? | Yes (go to Q45) | | | | | |  | 1 |
|  |  | No (go to Q65) | | | | | |  | 2 |
|  |  | Don’t recall (go to Q65) | | | | | |  | 9 |
|  |  |  | | | | | |  |  |
|  | In the 3 months *[or as appropriate]* before coming into prison how often did you inject drugs? | Less than monthly | | | | | |  | 1 |
|  |  | Monthly or more often | | | | | |  | 2 |
|  |  | Weekly or more often | | | | | |  | 3 |
|  | *[Interviewer: check that “daily” and “ more than* | Daily | | | | | |  | 4 |
|  | *once a day” are distinguished]* | More than once a day | | | | | |  | 5 |
|  |  | Don’t recall | | | | | |  | 9 |
|  |  |  | | | | | |  |  |
|  | In the 3 months *[or as appropriat*e*]* before coming into prison, compared to the rest of your life, has your injecting pattern been…  *[Interviewer: assess the lifetime pattern of* | Stable | | | | | |  | 1 |
|  |  | Increasing | | | | | |  | 2 |
|  |  | Decreasing | | | | | |  | 3 |
|  |  | Don’t recall | | | | | |  | 9 |
|  | *injecting and code yes if frequency, sharing behaviours or drug of choice have changed*] | | | | | | | |  |
|  |  |  | | | | | |  |  |
|  | In the 3 months *[or as appropriate]* before coming to prison which drugs did you inject? *[Interviewer: read the options and emphasize on “injecting”]* |  | | | | | |  |  |
|  |  |  |  |  |  |  |  |  |  |
|  |  | Heroin | | | | | |  | 1 |
|  |  | Buprenorphine /Methadone | | | | | |  | 2 |
|  | Crystal meth/shabu/ice/goey/Amphetamine/Speed/methamphetamine | | | | | | |  | 3 |
|  | GHB/GBH/ liquid e/fantasy | | | | | | |  | 4 |
|  | *[Interviewer: Exclude medications prescribed* | Cocaine/ Coke | | | | | |  | 5 |
|  | *and administered by health workers,* | Benzodiazepines/Benzos | | | | | |  | 6 |
|  | *e.g. morphine in hospital]* | Anabolic/Steroids | | | | | |  | 7 |
|  | Other opiates/morphine/pethidine/omnopon | | | | | | |  | 8 |
|  | Hallucinogens/LSD/ Acid/Magic/Mushies/Daitura | | | | | | |  | 9 |
|  |  | Ecstacy/ E/MDA/MDMA | | | | | |  | 10 |
|  |  | Ketamine | | | | | |  | 11 |
|  |  |  | | | | | |  |  |
|  | In the 3 months *[or as appropriate]* before coming into prison has someone else injected you with drugs? (Given you a hit?) | Yes | | | | | |  | 1 |
|  |  | No | | | | | |  | 2 |
|  |  | Don’t recall | | | | | |  | 9 |
|  |  |  | | | | | |  |  |
|  | In the 3 months *[or as appropriate]* before coming into prison have you shared any part of the injecting equipment? That includes the needle, syringe, spoon, swabs, filters, mix or tourniquet? | Yes | | | | | |  | 1 |
|  |  | No | | | | | |  | 2 |
|  |  | Don’t recall | | | | | |  | 9 |
|  |  |  |  |  |  |  |  |  |  |
|  |  |  |  |  |  |  |  |  |  |
|  | [*Interviewer: mention that this includes sharing with their partner, and even once is a “yes”*] | | | | | | | | |
|  |  |  | | | | | |  |  |
|  | In the 3 months *[or as appropriate]* before coming into prison did you use injecting equipment that was not new and sterile? [*Interviewer: include the needle, syringe, spoon, swabs, filter, mix*] | Yes | | | | | |  | 1 |
|  |  | No | | | | | |  | 2 |
|  |  | Don’t recall | | | | | |  | 9 |
|  |  |  |  |  |  |  |  |  |  |
|  |  |  |  |  |  |  |  |  |  |
|  |  |  | | | | | |  |  |
|  | In the 3 months *[or as appropriate]* before coming into prison did you use injecting equipment after someone else had used it? | Yes (go to 52) | | | | | |  | 1 |
|  |  | No (go to 61) | | | | | |  | 2 |
|  |  | Don’t recall (go to 61) | | | | | |  | 9 |
|  |  |  | | | | | |  |  |
|  | In the 3 months *[or as appropriate]* before coming into prison how often did you use equipment after someone else had used it? | Less than monthly | | | | | |  | 0 |
|  |  | Monthly or more often | | | | | |  | 1 |
|  |  | Weekly or more often | | | | | |  | 2 |
|  | *[Interviewer: check that “daily” and “ more than* | Daily | | | | | |  | 3 |
|  | *once a day” are distinguished]* | More than once a day | | | | | |  | 4 |
|  | Don’t know but did use after someone else | | | | | | |  | 5 |
|  |  | Don’t recall | | | | | |  | 9 |
|  |  |  | | | | | |  |  |
|  | In the 3 months *[or as appropriate]* before coming into prison which equipment did you share? | | | | | | | |  |
|  |  | Yes | | | No | Don’t know | | | |
|  | Needle and syringe |  | | 1 |  | 2 |  | | 9 |
|  | Spoon |  | | 1 |  | 2 |  | | 9 |
|  | Mix |  | | 1 |  | 2 |  | | 9 |
|  | Filter |  | | 1 |  | 2 |  | | 9 |
|  | Swab |  | | 1 |  | 2 |  | | 9 |
|  | Tourniquet |  | | 1 |  | 2 |  | | 9 |
|  | Other |  | | 1 |  | 2 |  | | 9 |
|  |  |  | | | | | |  |  |
|  | In the 3 months *[or as appropriate]* before coming into prison did you attempt to bleach the shared equipment in any way? | Yes, always | | | | | |  | 1 |
|  |  | Yes, sometimes | | | | | |  | 2 |
|  |  | Never | | | | | |  | 3 |
|  |  | Don’t recall | | | | | |  | 9 |
|  |  |  | | | | | |  |  |
|  | In the 3 months *[or as appropriate]* before coming into prison did someone else help you inject? | Yes, always | | | | | |  | 1 |
|  |  | Yes, sometimes | | | | | |  | 2 |
|  |  | Never | | | | | |  | 3 |
|  | [*Interviewer: do not include medical injections*] | Don’t recall | | | | | |  | 9 |
|  |  |  | | | | | |  |  |
|  | In the 3 months *[or as appropriate]* before coming into prison did you help someone else to inject drugs? | Yes | | | | | |  | 1 |
|  |  | No | | | | | |  | 2 |
|  |  | Don’t recall | | | | | |  | 9 |
|  |  |  | | | | | |  |  |
|  | In the 3 months *[or as appropriate]* before coming into prison how often did you use a needle and syringe exchange? | Never | | | | | |  | 0 |
|  |  | Some times | | | | | |  | 1 |
|  |  | Most times I needed | | | | | |  | 2 |
|  |  | Every time I needed | | | | | |  | 3 |
|  |  |  | | | | | |  |  |
|  | In the 3 months *[or as appropriate]* before coming into prison did you inject in the Medically Supervised Injecting Centre? | Never | | | | | |  | 0 |
|  |  | Less than monthly | | | | | |  | 1 |
|  |  | Monthly or more often | | | | | |  | 2 |
| [*Interviewer: MSIC is in Kings Cross, NOT shooting galleries]* Weekly or more often | | | | | | | |  | 3 |
| *[Interviewer: check that “daily” and “ more than once a day” are distinguished]* Daily | | | | | | | |  | 4 |
|  |  | More than once a day | | | | | |  | 5 |
|  |  | Don’t recall | | | | | |  | 9 |
|  |  |  | | | | | |  |  |
| ***VII. THIS SECTION IS ABOUT INJECTING DRUG USE SINCE ENTRY INTO PRISON THIS TIME*** | | | | | | | | | |
|  |  |  | | | | | |  |  |
|  | Since coming into prison have you injected drugs? | Yes (go to Q66) | | | | | |  | 1 |
|  |  | No (go to Q84) | | | | | |  | 2 |
|  |  | Don’t recall (go to Q84) | | | | | |  | 9 |
|  | Since coming into prison how often have you injected? | Less than monthly | | | | | |  | 1 |
|  |  | Monthly or more often | | | | | |  | 2 |
|  | *[Interviewer: check that “daily” and “ more than* | Weekly or more often | | | | | |  | 3 |
|  | *once a day” are distinguished]* | Daily | | | | | |  | 4 |
|  |  | More than once a day | | | | | |  | 5 |
|  |  | Don’t recall | | | | | |  | 9 |
|  |  |  | | | | | |  |  |
|  | Since coming into prison this time compared to the rest of your life, has your injecting pattern been…  *[Interviewer: assess the lifetime pattern of injecting* | Stable | | | | | |  | 1 |
|  |  | Increasing | | | | | |  | 2 |
|  |  | Decreasing | | | | | |  | 3 |
|  |  | Don’t recall | | | | | |  | 9 |
|  | *and code yes if frequency, sharing behaviour or drug of choice have changed]* | | | | | | | | |
|  |  | | | | | | |  |  |
|  | Since coming to prison which drug/s have you injected? *[interviewer: read the options and emphasize on “injecting”]* |  | | | | | |  |  |
|  |  | Heroin | | | | | |  | 1 |
|  |  | Buprenorphine/Methadone | | | | | |  | 2 |
|  | Crystal meth/shabu/ice/goey/Amphetamine/Speed/methamphetamine | | | | | | |  | 3 |
|  | GHB/GBH/ liquid e/fantasy | | | | | | |  | 4 |
|  | *[interviewer: Exclude medications prescribed* | Cocaine/ Coke | | | | | |  | 5 |
|  | *and administered by health workers,* | Benzodiazepines/Benzos | | | | | |  | 6 |
|  | *e.g. Morphine in hospital]* | Anabolic/Steroids | | | | | |  | 7 |
|  | Other opiates/morphine/pethidine/omnopon | | | | | | |  | 8 |
|  | Hallucinogens/LSD/ Acid/Magic/Mushies/Daitura | | | | | | |  | 9 |
|  |  | Ecstacy/ E/MDA/MDMA | | | | | |  | 10 |
|  |  | Ketamine | | | | | |  | 11 |
|  |  |  | | | | | |  |  |
|  | Since coming into prison has someone else ever injected you the drug? (Given you a hit?) | Yes | | | | | |  | 1 |
|  |  | No | | | | | |  | 2 |
|  |  | Don’t recall | | | | | |  | 9 |
|  |  |  | | | | | |  |  |
|  | Since coming into prison have you ever shared any part of the injecting equipment - that includes the needle, syringe, spoon, swabs, filters, mix or tourniquet? | Yes | | | | | |  | 1 |
|  |  | No | | | | | |  | 2 |
|  |  | Don’t recall | | | | | |  | 9 |
|  |  |  |  |  |  |  |  |  |  |
|  |  |  | | | | | |  |  |
|  | Since coming into prison did you use injecting equipment that was not new and sterile? | Yes | | | | | |  | 1 |
|  |  | No | | | | | |  | 2 |
|  |  | Don’t recall | | | | | |  | 9 |
|  |  |  | | | | | |  |  |
|  | Since coming into prison did you ever use injecting equipment after someone | Yes (go to 73) | | | | | |  | 1 |
|  |  | No (go to 82) | | | | | |  | 2 |
|  | else had used it? | Don’t recall (go to 82) | | | | | |  | 9 |
|  |  |  | | | | | |  |  |
|  | Since coming into prison how often did you use equipment after someone else had | Never | | | | | |  | 0 |
|  |  | Some times | | | | | |  | 1 |
|  | used it? | Most times I injected | | | | | |  | 2 |
|  |  | Every time I injected | | | | | |  | 3 |
|  |  |  | | | | | |  |  |
|  | Since coming into prison which equipment did you share? | | | | | | | |  |
|  |  | Yes | | | No | Don’t know | | | |
|  | Needle and syringe |  | 1 | |  | 2 |  | | 9 |
|  | Spoon |  | 1 | |  | 2 |  | | 9 |
|  | Mix |  | 1 | |  | 2 |  | | 9 |
|  | Filter |  | 1 | |  | 2 |  | | 9 |
|  | Swab |  | 1 | |  | 2 |  | | 9 |
|  | Tourniquet |  | 1 | |  | 2 |  | | 9 |
|  | Other |  | 1 | |  | 2 |  | | 9 |
|  |  |  | | | | | |  |  |
|  | Since coming into prison did you attempt to bleach the shared equipment in any way since coming into prison? | Yes, always | | | | | |  | 1 |
|  |  | Yes, sometimes | | | | | |  | 2 |
|  |  | Never | | | | | |  | 3 |
|  |  | Don’t recall | | | | | |  | 9 |
|  |  |  | | | | | |  |  |
|  | Since coming into prison did someone else help you inject? | Yes, always | | | | | |  | 1 |
|  |  | Yes, sometimes | | | | | |  | 2 |
|  |  | Never | | | | | |  | 3 |
|  |  | Don’t recall | | | | | |  | 9 |
|  |  |  | | | | | |  |  |
|  | Since coming into prison did you help someone else to inject drugs? | Yes | | | | | |  | 1 |
|  |  | No | | | | | |  | 2 |
|  |  | Don’t recall | | | | | |  | 9 |
|  |  |  | | | | | |  |  |
|  |  |  | | | | | |  |  |
|  |  |  | | | | | |  |  |
| *VIII. THIS SECTION IS ABOUT DRUG TREATMENT* | | | | | | | |  |  |
|  |  |  | | | | | |  |  |
|  | Have you ever seen a Drug and Alcohol counselor or received counseling for a drug problem? | Yes | | | | | |  | 1 |
|  |  | No | | | | | |  | 2 |
|  |  | Don’t recall | | | | | |  | 9 |
|  | *[Interviewer: at least one complete session of counseling]* |  | | | | | |  |  |
|  |  |  | | | | | |  |  |
|  | Are you currently on a methadone program? | Yes (go to 86) | | | | | |  | 1 |
|  |  | No (go to 89) | | | | | |  | 2 |
|  |  | Don’t recall (go to 89) | | | | | |  | 9 |
|  |  |  | | | | | |  |  |
|  | What is your current dose of Methadone | ______________ mg | | | | | |  |  |
|  | (1ml = 5mg) [*Interviewer: 4ml= 20mg = less than a tea spoon. Make sure to clarify the* | | | | | | |  |  |
|  | *dose, if not clear , check the medical record. A typical dose range is 20-100mg*]*.* | | | | | | |  |  |
|  |  | | | | | | |  |  |
|  | In the last month has your Methadone dose been: | Stable | | | | | |  | 1 |
|  |  | Increasing | | | | | |  | 2 |
|  |  | Decreasing | | | | | |  | 3 |
|  |  | Don’t recall | | | | | |  | 9 |
|  |  |  | | | | | |  |  |
|  | How many days have you missed your methadone since being arrested? | __________________ | | | | | |  |  |
|  |  | Never | | | | | |  | 1 |
|  |  | Don’t recall | | | | | |  | 9 |
|  |  |  | | | | | |  |  |
|  | Are you currently on a Buprenorphine program? | Yes (go to 90) | | | | | |  | 1 |
|  |  | No ( End of interview ) | | | | | |  | 2 |
|  | Don’t recall (End of Interview) | | | | | | |  | 9 |
|  |  |  | | | | | |  |  |
|  | What is your current dose of Buprenorphine? [tablets (0.4mg,2mg,8mg)] | ______________mg | | | | | |  |  |
|  | [*Interviewer: typical dose range is 2-16mg*] |  | | | | | |  |  |
|  |  |  | | | | | |  |  |
|  | In the last month has your Buprenorphine dose been: | Stable | | | | | |  | 1 |
|  |  | Increasing | | | | | |  | 2 |
|  |  | Decreasing | | | | | |  | 3 |
|  |  | Don’t recall | | | | | |  | 9 |
|  |  |  | | | | | |  |  |
|  | How many days have you missed your Buprenorphine since being arrested? | __________________ | | | | | |  |  |
|  |  | Never | | | | | |  | 1 |
|  |  | Don’t recall | | | | | |  | 9 |
|  |  |  | | | | | |  |  |
|  | | | | | | | | | |
