# Supplementary material for: Incidence of primary hepatitis C infection and risk factors for transmission in an Australian prisoner cohort
Source: BMC Public Health. 2010 Oct 22;10:633. doi: 10.1186/1471-2458-10-633 (PMC2975656; doi:10.1186/1471-2458-10-633)
Supplement: Additional file 2 — IDU behaviors amongst inmates receiving MMT. Table of frequencies and univariate comparisons [file 1471-2458-10-633-S2.DOC]

**Table 5 IDU behaviors amongst inmates receiving MMT who became incident cases or remained uninfected in comparison to inmates not receiving MMT in the HITS cohort (n=488**).

| **Variable** | **A. Receiving MMT**  **No. (%)** | | **B. Not receiving MMT**  **No. (%)** | |  |  |
| --- | --- | --- | --- | --- | --- | --- |
|  | **C. Non-cases**  **No. (%)**  **(n=63)** | **D. Incident cases No. (%)**  **(n=36)** | **Non-cases**  **No. (%)**  **(n=333)** | **Incident cases No. (%)**  **(n=58)** | ***p***  **(A vs B)** | ***p***  **(C vs D)** |
|  |  |  |  |  |  |  |
| Ever daily IDU | 55 (87) | 34 (94) | 241 (72) | 48 (83) | **0.001** | 0.32 |
| Ever shared IDU equipment | 43 (68) | 22 (61) | 198 (61) | 41 (71) | 0.41 | 0.47 |
| Ever injected methadone / buprenorphine | 33 (52) | 18 (50) | 92 (28) | 20 (34) | **<0.001** | 0.82 |
| 3 months before incarceration IDU * | 19 (76) | 21 (72) | 96 (65) | 37 (82) | 0.50 | 0.76 |
| 3 months before incarceration ‘stable’ pattern of IDU * | 5 (20) | 7 (24) | 8 (18) | 19 (13) | 0.15 | 0.72 |
| 3 months before incarceration shared IDU equipment * | 9 (36) | 7 (24) | 32 (22) | 11 (24) | 0.27 | 0.34 |
| Since imprisonment IDU | 24 (38) | 11 (31) | 78 (24) | 20 (35) | **0.04** | 0.45 |
| Since imprisonment ‘decreasing’ pattern of IDU | 20 (32) | 11 (31) | 61 (18) | 16 (28) | **0.01** | 0.90 |
| Since imprisonment shared IDU equipment | 23 (37) | 8 (22) | 70 (21) | 19 (33) | 0.08 | 0.14 |
| Since imprisonment injected methadone / buprenorphine | 12 (19) | 2 (6) | 47 (14) | 12 (21) | 0.81 | 0.06 |

* Only examined in inmates who were not continuously in prison during the period of observation (Receiving MMT: Non cases n= 29, Incident cases n= 25; Not receiving MMT: Non cases n = 147, Incident cases n= 45)
